# Driving Under the Influence of Drugs: A Single Parallel Monitoring-Based Quantification Approach on Whole Blood

**DOI:** 10.3389/fchem.2020.00626

**Published:** 2020-08-26

**Authors:** Timothée Joye, Katell Rocher, Julien Déglon, Jonathan Sidibé, Bernard Favrat, Marc Augsburger, Aurélien Thomas

**Affiliations:** ^1^Forensic Toxicology and Chemistry Unit, CURML, Lausanne University Hospital, Geneva University Hospitals, Lausanne, Switzerland; ^2^Faculty Unit of Toxicology, CURML, Faculty of Biology and Medicine, University of Lausanne, Lausanne, Switzerland; ^3^Unit of Medicine and Traffic Psychology, CURML, Lausanne University Hospital, Geneva University Hospitals, Lausanne, Switzerland; ^4^Center for Primary Care and Public Health (Unisanté), University of Lausanne, Lausanne, Switzerland

**Keywords:** parallel reaction monitoring, quantitative analysis, whole blood, driving under the influence of drugs, multi-analyte

## Abstract

Driving under the influence of psychoactive substances is a major cause of motor vehicle crashes. The identification and quantification of substances most frequently involved in impaired-driving cases in a single analytic procedure could be an important asset in forensic toxicology. In this study, a highly sensitive and selective liquid chromatography (LC) approach hyphenated with Orbitrap high-resolution mass spectrometry (HRMS) was developed for the quantification of the main drugs present in the context of driving under the influence of drugs (DUID) using 100 μL of whole blood. This procedure involves a simple sample preparation and benefit from the selectivity brought by parallel reaction monitoring (PRM) allowing to solve most DUID cases using a single multi-analyte injection. The method was fully validated for the quantification of the major classes of psychoactive substances associated with impaired-driving (cannabinoids, cocaine and its metabolites, amphetamines, opiates and opioids, and the major benzodiazepines and z-drugs). The validation guidelines set by the “Société Française des Sciences et des Techniques Pharmaceutiques” (SFSTP) were respected for 22 psychoactive substances using 15 internal standards. Trueness was measured to be between 95.3 and 107.6% for all the tested concentrations. Precision represented by repeatability and intermediate precision was lower than 12% while recovery (RE) and matrix effect (ME) ranged from 49 to 105% and from −51 to 3%, respectively. The validated procedure provides an efficient approach for the simultaneous and simple quantification of the major drugs associated with impaired driving benefiting from the selectivity of PRM.

## Introduction

Road crashes are a worldwide public health issue, causing a significant number of deaths and injuries each year. Indeed, 1.25 million people died and about 50 million were injured in road traffic crashes in 2015 according to the World Health Organization (2015). In addition, in Europe almost 25% of adults reported at least one instance of illicit drug consumption in their life (Liakoni et al., 2018). These two issues are closely linked, since one of the major causes of road crashes is the consumption of psychotropic substances, including drugs and alcohol, resulting in driving impairment (Elliott et al., 2009; Favretto et al., 2018). For instance, in Norway, at least 21% of traffic crashes were related to either alcohol or drug use between 2005 and 2015 (Valen et al., 2019). The total number of victims of fatal crashes has significantly decreased in the past years in Western countries thanks to efficient prevention. Yet, the use of medicinal or illicit drug and/or alcohol is an increasing phenomenon in Europe (Snenghi et al., 2018; Pelletti et al., 2019), and the percentage of fatal crashes due to the driver's impairment remain constant (between 17 and 22% from 1995 to 2017) in Switzerland [Office Fédéral de la Statistique (OFS), 2018].

Due to the large variety of drugs and pharmaceuticals with various psychoactive effects, there is a need for medical experts to establish solid statement on a potential driving-impairment and for official quantification of drugs and alcohol levels in blood (Martin et al., 2017). In Switzerland, a zero tolerance with technical cut-offs is implemented regarding classical drugs of abuse (DoA) toward drivers (1.5 ng/ml for THC and 15 ng/ml for morphine, cocaine, amphetamine, methamphetamines, MDEA, and MDMA) (Walsh et al., 2004; Steuer et al., 2016). The situation is more complex regarding the consumption of medicinal drugs and the toxicological interpretation of their concentration (Ravera et al., 2012). With respect to the law, the driving capability under pharmaceuticals is concomitantly determined by a “three pillars expertise” including police assessment, medical expertise, and toxicological analysis in blood, being the biological matrix of reference regarding toxicological interpretation (Steuer et al., 2014). The Swiss Federal Roads Office (FEDRO) defines a list of controlled substances that the laboratories must be able to quantify in the context of external quality controls (EQCs) in whole blood regarding driving under the influence of drugs (DUID). Those recommendations, associated with the knowledge of drug prevalence among suspected drivers, were used to establish a list of substances of interest in the present study.

Improvements regarding instrumentation, notably brought on by the developments of Orbitrap technology, offer new opportunities in terms of analytical strategies (Hoffman et al., 2018; Joye et al., 2019). Indeed, various Orbitrap-based parallel reaction monitoring (PRM) applications have been reported, especially in the field of proteomics (Domon and Gallien, 2015; Rauniyar, 2015; Bourmaud et al., 2016). In a PRM acquisition, a precursor selected by a quadrupole is fragmented in a higher-energy collisional dissociation (HCD) cell (Ronsein et al., 2015). Following this experiment, all product ions are simultaneously acquired in the high-resolution Orbitrap analyzer. Up to now, the use of triple-quadrupole (QQQ) using Selected Reaction Monitoring (SRM) has been the gold standard regarding targeted quantitative analyses (Hopfgartner et al., 2004; Rauniyar, 2015). However, SRM and PRM have comparable sensitivity with similar linearities, dynamic ranges, precision, and repeatability (Domon and Gallien, 2015; Joye et al., 2020). Yet Orbitrap-based PRM offers further advantages, since the acquisition of all selected precursors' fragments is performed, thereby limiting the *a priori* information required for method development. Indeed, the selection of quantifying ions is only necessary during the data processing step once the whole fragmentation spectra is acquired. Moreover, HRMS provides a higher specificity, allowing for the separation of the background ions from the targeted molecules (Ronsein et al., 2015).

Drug quantification can easily benefit from the PRM specificities that have been enlightened for proteomic applications. Even though this strategy is relatively recent regarding illicit drug and pharmaceuticals analyses in toxicology, it has received a growing interest. Indeed, PRM quantification has been reported for the quantitative analysis of abiraterone (Bhatnagar et al., 2018), beclabuvir (Jiang et al., 2017), anticoagulant rodenticides (Gao et al., 2018), and sterols (Schott et al., 2018). Regarding drugs of abuse, a first application has been described for the quantification of cannabinoids in whole blood (Joye et al., 2020).

Herein, we present a validated single multi-analyte procedure for the quantification of the main substances regarding DUID cases using 100 μL of whole blood. The quantified substances were selected based on the FEDRO list and the prevalence of substances consumed by the drivers in Switzerland (Augsburger and Rivier, 1997; Augsburger et al., 2005; Senna et al., 2010). The validated approach uses the advantages provided by HRMS and especially PRM for the simultaneous quantification of 22 DoA and pharmaceuticals alongside 15 internal standards (IS), enabling the solving of most DUID cases with a single injection and a simple sample preparation.

## Materials and Methods

### Standards and Reagents

Water, methanol, formic acid (FA), and ammonium formiate were furnished by Biosolve. Drugs standard were purchased from Cerilliant or Lipomed, at either 1 mg/ml or 100 μg/ml. External quality controls (ECQ) were purchased from Medidrug, ACQ Science, or Clincheck. Blank lyophilized whole blood was acquired from ACQ Science.

### Solution Preparation

Standard solutions containing tetrahydrocannabinol (THC), 11-Nor-9-carboxy-THC (THC-COOH), alprazolam, amphetamine, methamphetamine, 3,4-methylendioxymethamphetamine (MDMA), 3,4-methylene dioxy-amphetamine (MDA), methylphenidate, cocaine, cocaethylene, lorazepam, bromazepam, zolpidem, benzoylecgonine, morphine, codeine, methadone, tramadol, O-desmethyltramadol, diazepam, nordiazepam, oxazepam were prepared for calibration curve and internal quality control (IQC) preparation. In parallel, solutions containing THC-D3, THC-COOH-D9, cocaine-D3, benzoylecgonine-D3, amphetamine-D8, MDMA-D5, methylphenidate-D10, morphine-D3, codeine-D3, methadone-D3, tramadol^13^C-D3, O-desmethyltramadol-D6, nordiazepam-D5, alprazolam-D5, and zolpidem-D6 were prepared as internal standard (IS) solutions.

Calibration samples were prepared by spiking lyophilized whole blood at 5 concentration levels (Table 1). IS were added to reach a final concentration of 10 (THC-D3), 100, or 1,000 ng/ml depending on the specific calibration range.

**Table 1:** Calibration levels and quantification parameters (IS, polarity, parent ion m/z, and quantifier ions) for the substances of interest. The calibration ranges are in adequacy with the legal thresholds and the therapeutic ranges.

	**Calibration levels (ng/ml)**					**Quantification parameters**					
	**Level 1**	**Level 2**	**Level 3**	**Level 4**	**Level 5**	**Polarity**	**Parent Ion (m/z) → quantifier ion**	**Qualifier ion for data processing (m/z)**	**IS**	**IS concentration (ng/ml)**	**IS parent ion → quantifier ion**
THC	1	2	5	10	20	+	315.2319 → 193.1222	123.0440	THC-D3	10	318.2507 → 196.1413
THC-COOH	5	10	25	50	100	–	343.1915 → 245.1546	191.1068	THC-COOH-D9	100	352.2479 → 254.2108
Cocaine	10	20	50	100	200	+	304.1543 → 182.1177	82.0657	Cocaine-D3	100	307.1731 → 185.1364
Cocaethlyene	50	100	250	500	1,000	+	318.1699 → 196.1333	82.0651	Cocaine-D3	100	307.1731 → 185.1364
Benzoylecgonine	50	100	250	500	1,000	+	290.1387 → 168.1020	105.0338	Benzoylecgonine-D3	100	293.1575 → 171.1204
Amphetamine	10	20	50	100	200	+	136.1121 → 91.0547	119.0857	Amphetamine-D8	100	144.1623 → 97.0921
Methamphetamine	10	20	50	100	200	+	150.1277 → 91.0547	119.0857	Amphetamine-D8	100	144.1623 → 97.0921
MDA	10	20	50	100	200	+	180.1019 → 133.0648	105.0702	MDMA-D5	100	199.1489 → 165.0877
MDMA	10	20	50	100	200	+	194.1175 → 163.0753	135.0441	MDMA-D5	100	199.1489 → 165.0877
Methylphenidate	10	20	50	100	200	+	234.1488 → 84.0813	56.0503	Methylphenidate-D10	100	244.2116 → 93.1376
Morphine	5	50	500	1,000	2,000	+	286.1438 → 201.0908	229.0858	Morphine-D3	1,000	289.1626 → 201.0906
Codeine	5	50	500	1,000	2,000	+	300.1594 → 215.1061	58.0659	Codeine-D3	1,000	303.1783 → 215.1061
Methadone	5	50	500	1,000	2,000	+	310.2165 → 105.0339	219.1167	Methadone-D3	1,000	313.2354 → 105.0337
Tramadol	5	50	500	1,000	2,000	+	264.1958 → 58.0659	–	Tramadol-^13^C-D3	1,000	269.2287 → 58.0657
O-Desmethyltramadol	5	50	500	1,000	2,000	+	250.1801 → 58.0659	–	O-Desmethyltramadol-D6	1,000	256.2178 → 64.1033
Diazepam	100	200	500	1,000	2,000	+	285.0789 → 154.0417	193.0885	Nordiazepam-D5	1,000	276.0947 → 140.0258
Nordiazepam	100	200	500	1,000	2,000	+	271.0633 → 140.0262	165.0212	Nordiazepam-D5	1,000	276.0947 → 140.0258
Oxazepam	100	200	500	1,000	2,000	+	287.0582 → 241.0527	104.0498	Nordiazepam-D5	1,000	276.0947 → 140.0258
Lorazepam	20	50	100	150	300	+	321.0192 → 229.0527	163.0055	Alprazolam-D5	100	314.1215 → 286.1018
Bromazepam	20	50	100	150	300	+	316.0080 → 182.0839	209.0945	Alprazolam-D5	100	314.1215 → 286.1018
Alprazolam	5	10	25	50	100	+	309.0902 → 281.0707	274.1208	Alprazolam-D5	100	314.1215 → 286.1018
Zolpidem	40	100	200	300	600	+	308.1757 → 235.1230	263.1175	Zolpidem-D6	100	314.2134 → 235.1224

### Sample Pre-treatment

IS solutions were spiked in Eppendorfs and evaporated to dryness before adding 100 μL of whole blood. The extraction was then performed by protein precipitation using 300 μL of methanol. After centrifugation for 10 min at 14,000 rpm, the upper methanolic phase was transferred into a new Eppendorf and evaporated to dryness under a nitrogen flow. Reconstitution was performed using 100 μL of 1:9 methanol: water and 10 μL were injected into the LC-HRMS system (Supplemental Figure 1).

### LC-HRMS Method

A Thermo Scientific Ultimate 3000 LC system with a Phenomenex 2.6 μm C18 (10 cm × 2.1 mm) maintained at 45°C was used for chromatographic separation. Mobile phases were composed of A, ammonium formate 10 mM at pH 3.3, and B, methanol with 0.1% FA. Phase B was ramped linearly from 2 to 98% over 7.5 min. The column was then washed at 98% of B for 3.5 min, followed by a 6 min re-equilibration at 2% of B at 300 μL/min for a total analysis run of 17 min. The LC was coupled to a Q Exactive Plus system (Thermo Scientific, Bremen, Germany) via a heated electro spray ionization (ESI) source (H-ESI II probe, Thermo Scientific). The ionization spray voltage was set to 3 kV, sheath gas flowrate was set to 40, and auxiliary gas flowrate to 10 (both in arbitrary units). The method functioned in PRM, using an inclusion list containing the exact mass of the parent ion and the retention time windows for the different analytes. A polarity switch in negative was performed at 7.5 min for the specific detection of THC-COOH with a switch back in positive polarity at 8.7 min for the detection of THC. Resolution was set to 17,500 for the HCD fragmentation performed using an NCE at 50 eV with an AGC target of 1e5 and a maximum IT of 100 ms.

### Method Validation

The validation criteria used to evaluate the analytical process was based on the directives of the “Société Française des Sciences et des Techniques Pharmaceutiques” (SFSTP) regarding bioanalytical methods and adapted to our specific requirements (Boulanger et al., 2003; Peters et al., 2007; Lynch, 2016). Two product ions (one quantifier and one qualifier) were used for data processing (Table 1) and full MS/MS spectra were compared with the online advanced mass spectral database *m/z cloud*. The validation was performed over 3 non-consecutive days (*p* = 3). The trueness and precision were evaluated using a variance analysis-based statistical treatment (ANOVA). Calibration (Cal) was performed in duplicate at 5 different concentration levels (k = 5) (Table 1) while quality controls (QCs) were prepared in quadruplicate at the two lowest and highest concentration levels (k = 4). Using the acquired data, trueness, precision, accuracy, linearity, limits of detection (LOD), and quantification (LOQ) were determined. Six different blank bloods were analyzed for selectivity assessment investigating for potential interferences. The approach developed by Matuszweski et al. was used for recovery (RE) and matrix effect (ME) evaluation (Matuszewski et al., 2003). In this optic, three sample sets were prepared, including all the substances of interest at two concentration levels (low being level 2 and high being level 4 described in Table 1). Sample set 1 represented neat standards spiked after the extraction, while sample set 2 represented blank blood spiked after extraction. Sample set 3 represented blank blood spiked before extraction. The absence of interfering peaks at the established retention times (RT) for the analytes and the IS was used to ensure the specificity. The chemical stability of all analytes was evaluated under sample handling and storage conditions at low and high concentrations in five replicates. Benchtop (6 h, room temperature), autosampler (24 h, 5°C), three cycles of freeze-thaw (−20°C), and short term (1 week, −20°C) conditions were used for stability determination.

In order to evaluate the method, 8 different EQCs were analyzed in duplicates using the exact same procedure.

## Results and Discussion

### Method Development

**Figure 1:**
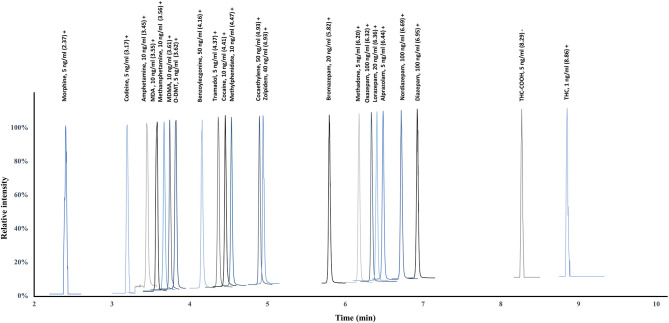
Chromatographic separation at LOQ (calibration level 1) using retention time windows to ensure a sufficient number of acquisition points for proper quantification.

In the present study, 22 analytes (15 IS) included in the main classes of drugs of abuse, as well as the major benzodiazepines, were analyzed using a single simultaneous multi-analyte quantitative approach. This list of substances was established based on the FEDRO recommendations and on the knowledge of the prevalence of psychoactive compounds among suspected impaired drivers. A nationwide study performed on 4,668 samples collected on suspected drivers in 2010 in Switzerland proved cannabinoids (48%), alcohol (35%), cocaine (25%), opiates (15%), amphetamines (7%), and benzodiazepines (6%) to be the most detected substances (Senna et al., 2010). The use of such multi-analyte approaches is challenging due to the various physico-chemical properties of the substances of interest and requires specific care during method development. To ensure a proper quantification, retention time windows were set for the acquisition ensuring the acquisition of a sufficient number of acquisition points (Figure 1). For good-quality integration and reproducible quantification, a minimum of 10–15 points is necessary to define exactly the peak start, peak apex, and peak end. The method was designed to resolve the wide majority of DUID cases using a single procedure and a limited amount of biological sample (Supplemental Figure 1) (Senna et al., 2010). The method allows the successful PRM-based quantification of cannabinoids, amphetamines, cocaine and its metabolites, opiates and opioids, and the major benzodiazepines at the sensitivity necessary for legal thresholds and therapeutic ranges (OOCCR-OFROU, 2008; Schulz et al., 2012).

### Trueness and Precision

**Figure 2:**
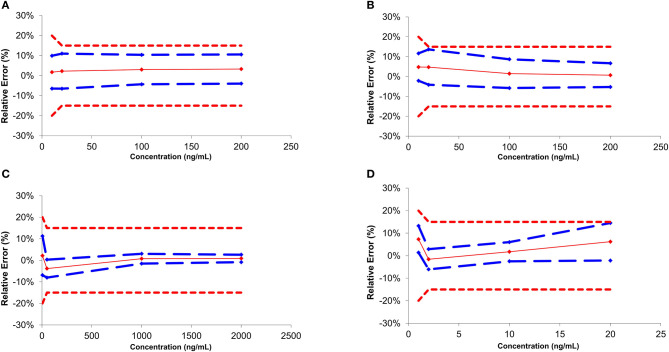
Accuracy profile for the main classes of drugs of abuse regulated by FEDRO [Cocaine **(A)**, Amphetamine **(B)**, Morphine **(C)**, and THC **(D)**].

Independent QC samples at 4 different calibration levels were injected in 4 replicates over 3 non-consecutive days for the determination of trueness and precision. Accuracy represents the total error and is divided into trueness (representing the “bias” or the systematic error) and precision (referring as the standard deviation or random errors) (Gonzalez et al., 2010). The trueness can be evaluated by calculating the percentage difference between the experimental and the expected theoretical values. In the present study, the systematic error varied from −4.7 to 7.6% (Table 2). Precision was divided into two parameters: the relative standard deviation (repeatability or R_R.S.D._) and the inter-day variability (intermediate precision or IP_R.S.D._). R_R.S.D._ represents the variability under similar conditions, meaning that the analyses are performed by the same operator using the same reagents and samples. On the other hand, IP_R.S.D._ represented the variability associated with the use of the same samples on different days with different reagents. Precision parameters were evaluated to be between 1.1 and 11.6% (Table 2). Accuracy profiles are visual representations combining both the trueness and the precision to represent the uncertainty measurement (Figure 2). Precision is represented by the calculated confidence limit at 95% at each concentration level. Accuracy profiles also include the representation of acceptance limits of ±20% at the LLOQ suggested for method validation (±15% at the other calibration levels). All analyzed QCs were within the acceptance limits.

**Table 2:** Results for trueness, precision, and linearity (k is the number of concentration levels, n the number of repetitions by levels, and p the number of non-consecutive days).

**Trueness (%) (k = 4;** ***n*** **= 4;** ***p*** **= 3)**				
**Calibration level (ng/ml)**	**Level 1**	**Level 2**	**Level 4**	**Level 5**
THC	107.3	98.4	101.8	106.2
THC-COOH	100.9	104.2	102.4	101.9
Cocaine	101.4	102.2	103.0	103.3
Cocaethlyene	102.1	106.7	101.1	99.4
Benzoylecgonine	101.8	103.6	100.6	99.8
Amphetamine	107.7	104.8	101.5	100.7
Methamphetamine	102.9	101.2	100.6	97.2
MDA	104.4	105.3	103.4	98.2
MDMA	107.2	102.2	97.6	98.3
Methylephenidate	104.8	103.4	102.4	100.6
Morphine	101.6	96.1	100.8	100.6
Codeine	103.5	107.6	103.2	102.4
Methadone	107.6	103.5	104.4	100.5
Tramadol	103.8	98.5	100.4	99.6
O-Desmethyltramadol	98.9	100.5	98.1	99.5
Diazepam	97.6	101.7	103.2	96.7
Nordazepam	98.4	102.6	103.0	99.6
Oxazepam	98.7	98.1	100.0	97.0
Lorazepam	101.0	99.5	99.3	102.2
Bromazepam	95.3	101.1	97.1	100.3
Alprazolam	100.9	105.1	101.8	97.2
**Repeatability/intermediate precision (RSD %) (k = 4**, ***n*** **= 4**, ***p*** **= 3)**				
**Calibration level (ng/ml)**	**Level 1**	**Level 2**	**Level 4**	**Level 5**
THC	5.6/5.6	3.1/3.9	3.9/4.0	3.2/7.1
THC-COOH	7.4/8.3	7.2/7.2	4.5/4.5	5.7/5.7
Cocaine	7.8/7.8	4.0/7.0	7.0/7.0	3.6/6.3
Cocaethlyene	3.6/5.7	4.7/5.0	2.2/2.3	4.5/4.5
Benzoylecgonine	5.0/5.0	2.4/3.3	2.2/2.2	3.1/3.5
Amphetamine	7.9/7.9	5.6/7.6	4.7/6.5	5.5/5.5
Methamphetamine	6.7/7.0	7.1/7.1	4.0/5.7	5.5/5.5
MDA	4.6/5.6	4.8/7.7	3.0/3.0	3.7/3.7
MDMA	5.2/6.9	6.8/6.8	4.8/4.8	6.4/4.7
Methylephenidate	3.6/4.1	4.1/5.6	3.1/3.7	3.8/3.8
Morphine	3.9/7.9	3.7/3.7	2.2/2.2	1.1/1.5
Codeine	7.2/8.4	5.3/6.3	5.0/6.2	2.8/4.8
Methadone	8.3/8.3	5.3/5.3	7.7/7.7	4.4/4.4
Tramadol	3.6/3.6	6.5/6.5	5.9/5.9	3.1/3.1
O-Desmethyltramadol	6.4/6.9	4.0/4.0	5.6/5.6	3.3/3.3
Diazepam	11.2/11.2	4.6/5.4	3.9/4.2	5.3/5.3
Nordazepam	3.8/4.2	3.1/3.2	2.2/2.2	1.9/2.2
Oxazepam	4.3/4.3	5.9/5.9	6.6/7.4	3.0/4.8
Lorazepam	8.4/11.0	5.5/5.5	9.4/9.8	4.6/6.5
Bromazepam	11.6/11.6	3.7/3.8	3.6/3.6	2.5/2.5
Alprazolam	9.0/9.0	5.2/5.6	7.0/7.0	3.8/4.2
**Linearity (k = 4, *n* = 4, *p* = 3)**				
	**Range (ng/ml)**	**Slope**	***R***^**2**^	**LOQ (ng/ml)**
THC	1–20	1.0623	0.9928	1
THC-COOH	5–100	1.0188	0.9946	5
Cocaine	10–200	1.0339	0.9933	10
Cocaethlyene	50–1,000	0.9905	0.9970	50
Benzoylecgonine	50–1,000	0.9959	0.9980	50
Amphetamine	10–200	1.0044	0.9937	10
Methamphetamine	10–200	0.9711	0.9944	10
MDA	10–200	0.9802	0.9967	10
MDMA	10–200	0.9779	0.9934	10
Methylephenidate	10–200	1.0046	0.9972	10
Morphine	5–2,000	1.0063	0.9995	5
Codeine	5–2,000	1.0237	0.9962	5
Methadone	5–2,000	1.0077	0.9951	5
Tramadol	5–2,000	0.9966	0.9978	5
O-Desmethyltramadol	5–2,000	0.9934	0.9977	5
Diazepam	5–2,000	0.9688	0.9936	5
Nordazepam	5–2,000	0.9969	0.9987	5
Oxazepam	5–2,000	0.9707	0.9945	5
Lorazepam	20–300	0.9921	0.9921	20
Bromazepam	20–300	1.0032	0.9980	20
Alprazolam	5–100	0.9697	0.9948	5
Zolpidem	40–600	1.0019	0.9983	40

### Linearity and LOQ

The definition of linearity stands as the method capacity to provide a result proportional to the actual sample concentration. To determine this parameter, a linear regression model based on the least square method was applied on the fit of the obtained concentration as a function of the theorical concentration. Slopes values were comprised between 0.9688 and 1.0623 with coefficients of determination above 0.9921 for all the compounds confirming the method linearity within the concentration ranges of interest (Table 2). LOQs were fixed according to the lowest point of the calibration curve (Table 1).

### Selectivity, Recovery, and Matrix Effect

Selectivity is defined as the ability to differentiate the analyte of interest from potential interferences. To assess the good selectivity of the method, six different blank blood samples were analyzed using the complete extraction procedure. No compounds impairing the detection and quantification of the analytes of interest were observed. HRMS technology offers a high selectivity due to its resolving power, therefore reducing the number of potential interferences (Chindarkar et al., 2014). However, ME, including ion suppression or enhancement, are often associated with the use of ESI as ion source challenging the method selectivity. The determination of such ME is therefore crucial to ensure a proper detection and quantification of the substances of interest. ME ranged from −51% (15% CV) of ion suppression for THC at low concentration and 3% (13% CV) of ion enhancement for lorazepam, being consistent with the existing literature (Table 3) (Simonsen et al., 2010; Fernandez Mdel et al., 2013; Montenarh et al., 2014; Steuer et al., 2014; Vaiano et al., 2016; De Boeck et al., 2017). All values concerning RE and ME are summarized in Table 3. To compensate for those undesirable ME, isotopically-labeled internal standards was used for normalization.

**Table 3:** Results for Recovery and Matrix Effect performed at low- and high-quality control concentrations.

**Matrix effect and recovery**				
	**ME low (CV %)**	**RE low (CV %)**	**ME high (CV %)**	**RE high (CV %)**
THC	−51% (15)	78% (18)	−34% (16)	81% (12)
THC-COOH	−7% (9)	57% (10)	−4% (3)	49% (4)
Cocaine	−25% (8)	92% (8)	−26% (8)	94% (10)
Cocaethlyene	−27% (6)	91% (6)	−21% (6)	95% (9)
Benzoylecgonine	−22% (4)	91% (6)	−19% (8)	92% (8)
Amphetamine	−10% (12)	96% (12)	−13% (7)	86% (12)
Methamphetamine	−12% (11)	76% (10)	−18% (12)	75% (10)
MDA	−32% (8)	106% (8)	−22% (5)	89% (7)
MDMA	−35% (10)	96% (9)	−18% (8)	95% (7)
Methylephenidate	−30% (8)	82% (17)	−25% (16)	81% (10)
Morphine	−23% (9)	96% (5)	−12% (4)	86% (17)
Codeine	−27% (4)	96% (16)	−13% (6)	95% (7)
Methadone	−21% (11)	94% (8)	−13% (10)	97% (9)
Tramadol	−27% (10)	88% (17)	−15% (6)	82% (19)
O-Desmethyltramadol	−29% (14)	81% (12)	−15% (8)	95% (13)
Diazepam	−30% (5)	73% (5)	−10% (11)	67% (10)
Nordazepam	0% (11)	82% (9)	−4% (9)	86% (9)
Oxazepam	−13% (6)	86% (7)	−5% (10)	82% (9)
Lorazepam	−7% (7)	80% (6)	3% (13)	95% (6)
Bromazepam	−18% (9)	96% (8)	−15% (10)	105% (7)
Alprazolam	−4% (12)	81% (6)	−1% (7)	84% (9)
Zolpidem	−16% (7)	73% (15)	−11% (13)	86% (12)

### Stability

Results regarding analytes' stability are listed in Table 4. Overall, stability ranged between 86 and 115%, assuring that the samples were stables within the tested conditions (auto-sampler, bench-top, 3 cycles of freeze-thaw and short-term stability.

**Table 4:** Three cycles of freeze-thaw (−20°C), benchtop (6 h, room temperature), autosampler (24 h, 5°C), and short-term (1 week, −20°C) conditions were performed in this stability assay at low- and high-quality control concentrations.

**Stability**								
	**Autosampler (5°C, 24 h)**		**Benchtop (Room Temp, 6 h)**		**Freeze-thaw (−20°C, 3 cycles)**		**Short term (−20°C, 1 week)**	
	**Low (CV %)**	**High (CV %)**	**Low (CV %)**	**High (CV %)**	**Low (CV %)**	**High (CV %)**	**Low (CV %)**	**High (CV%)**
THC	100% (13)	93% (3)	92% (7)	95% (5)	108% (11)	98% (2)	99% (17)	98% (4)
THC-COOH	103% (7)	97% (4)	106% (8)	97% (8)	100% (11)	101% (5)	101% (7)	99% (2)
Cocaine	109% (7)	95% (6)	99% (12)	92% (7)	104% (10)	99% (6)	108% (12)	96% (7)
Cocaethlyene	107% (12)	96% (6)	99% (12)	92% (16)	93% (6)	95% (14)	88% (9)	94% (8)
Benzoylecgonine	102% (7)	95% (4)	97% (11)	97% (6)	99% (9)	98% (3)	104% (7)	95% (4)
Amphetamine	100% (3)	95% (11)	100% (6)	96% (8)	95% (2)	96% (8)	93% (2)	91% (6)
Methamphetamine	103% (15)	100% (9)	95% (11)	97% (14)	92% (7)	103% (5)	105% (21)	96% (12)
MDA	104% (13)	98% (6)	95% (5)	86% (8)	101% (11)	101% (7)	109% (21)	96% (7)
MDMA	108% (10)	98% (5)	101% (6)	97% (5)	106% (10)	104% (6)	108% (16)	96% (6)
Methylephenidate	109% (21)	106% (13)	106% (10)	92% (19)	105% (10)	106% (16)	109% (20)	108% (16)
Morphine	91% (2)	98% (4)	94% (8)	100% (14)	90% (7)	104% (4)	86% (8)	98% (3)
Codeine	108% (10)	104% (6)	97% (5)	106% (7)	104% (8)	110% (9)	103% (9)	105% (9)
Methadone	106% (6)	98% (17)	96% (6)	101% (14)	99% (6)	105% (5)	102% (4)	98% (3)
Tramadol	108% (11)	98% (7)	102% (6)	100% (7)	98% (9)	105% (8)	100% (10)	98% (9)
O-Desmethyltramadol	99% (10)	101% (6)	98% (9)	101% (9)	99% (6)	106% (11)	96% (9)	94% (7)
Diazepam	104% (6)	97% (6)	101% (8)	88% (7)	90% (6)	96% (9)	102% (5)	90% (9)
Nordazepam	107% (4)	103% (4)	105% (7)	104% (3)	94% (7)	102% (5)	99% (7)	97% (5)
Oxazepam	102% (4)	99% (3)	95% (9)	95% (6)	86% (9)	107% (5)	113% (8)	109% (10)
Lorazepam	97% (16)	98% (6)	85% (7)	93% (11)	94% (10)	108% (6)	110% (8)	106% (8)
Bromazepam	98% (10)	95% (4)	91% (3)	91% (6)	104% (17)	97% (3)	97% (7)	98% (4)
Alprazolam	106% (9)	97% (11)	96% (6)	90% (8)	99% (9)	95% (8)	104% (9)	100% (6)
Zolpidem	109% (10)	93% (9)	92% (8)	93% (10)	115% (10)	97% (6)	97% (7)	94% (6)

### External Quality Control Analysis

**Figure 3:**
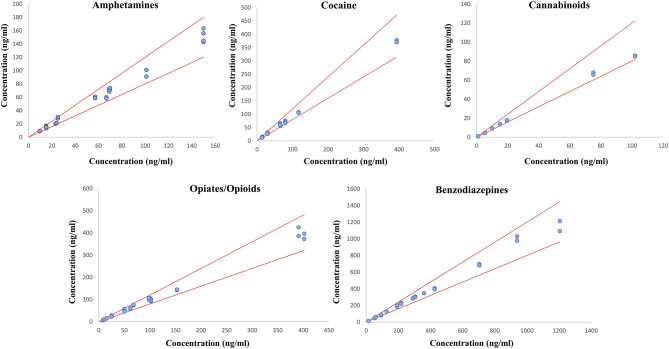
Method evaluation by comparing measured values and targeted commercial EQCs values. PRM measured concentrations are plotted as a function of the expected EQCs concentrations. The red lines represent the ±20% tolerance limits.

Eight commercial EQCs were analyzed in duplicates to ensure the robustness of the developed method and procedure. In total, the quantification was performed on 28 samples of amphetamines, 24 samples of cocaine and its metabolites, 16 samples of cannabinoids, 36 samples of opioids and opiates, and 28 samples of benzodiazepines and z-drugs. Results comparison between the described method and the expected EQCs values are represented in Figure 3 for the different classes of molecules involved in DUID cases. A good correlation was observed between the expected and the obtained values. The relative standard deviation was lower than 20% for all tested substances, confirming the efficiency of the PRM quantitative acquisition mode for toxicological analyses. This method confirms the potential of PRM as a solid alternative to classical MRM approaches (Li et al., 2016; Lv et al., 2018; Joye et al., 2020).

## Conclusion

A quick and efficient multi-analyte procedure was successfully developed in whole blood for the simultaneous quantification of 37 substances of interest in DUID cases. PRM represents an interesting alternative to classical MRM quantitative analyses, with the capability of precisely quantifying a large panel of substances with similar performance in terms of linearity, dynamic range, precision, and repeatability (Rauniyar, 2015). PRM quantification does not require *a priori* selection of the fragments of interest, leading to a simplified method development and better control over the quantification experiment, especially regarding multi-analyte approaches. The quantitative PRM procedure presented herein benefits the increased selectivity and sensitivity brought by HRMS, offering a clear alternative for quantitative toxicological analyses.

## Data Availability Statement

The raw data supporting the conclusions of this article will be made available by the authors, without undue reservation.

## Author Contributions

TJ supervised the project, participated in the design of the study, did several analyses, and wrote the manuscript. KR did most of the analytical work and participated to the reflexion toward the project. JD paritcipated in the strategic choices toward the study and its design and critically revised the manuscript. JS participated in the supervision and design of the project and critically revised the manuscript. BF participated in the design of the study and critically revised the manuscript. MA participated in the strategic choices toward the study and its design and critically revised the manuscript. AT is the main contributor to the study design, supervised the project, and critically revised the manuscript. All authors contributed to the article and approved the submitted version.

## Conflict of Interest

The authors declare that the research was conducted in the absence of any commercial or financial relationships that could be construed as a potential conflict of interest.
