# Supplementary figures and images for: Driving Under the Influence of Drugs: A Single Parallel Monitoring-Based Quantification Approach on Whole Blood
Source: Front Chem. 2020 Aug 26;8:626. doi: 10.3389/fchem.2020.00626 (PMC7480261; doi:10.3389/fchem.2020.00626)

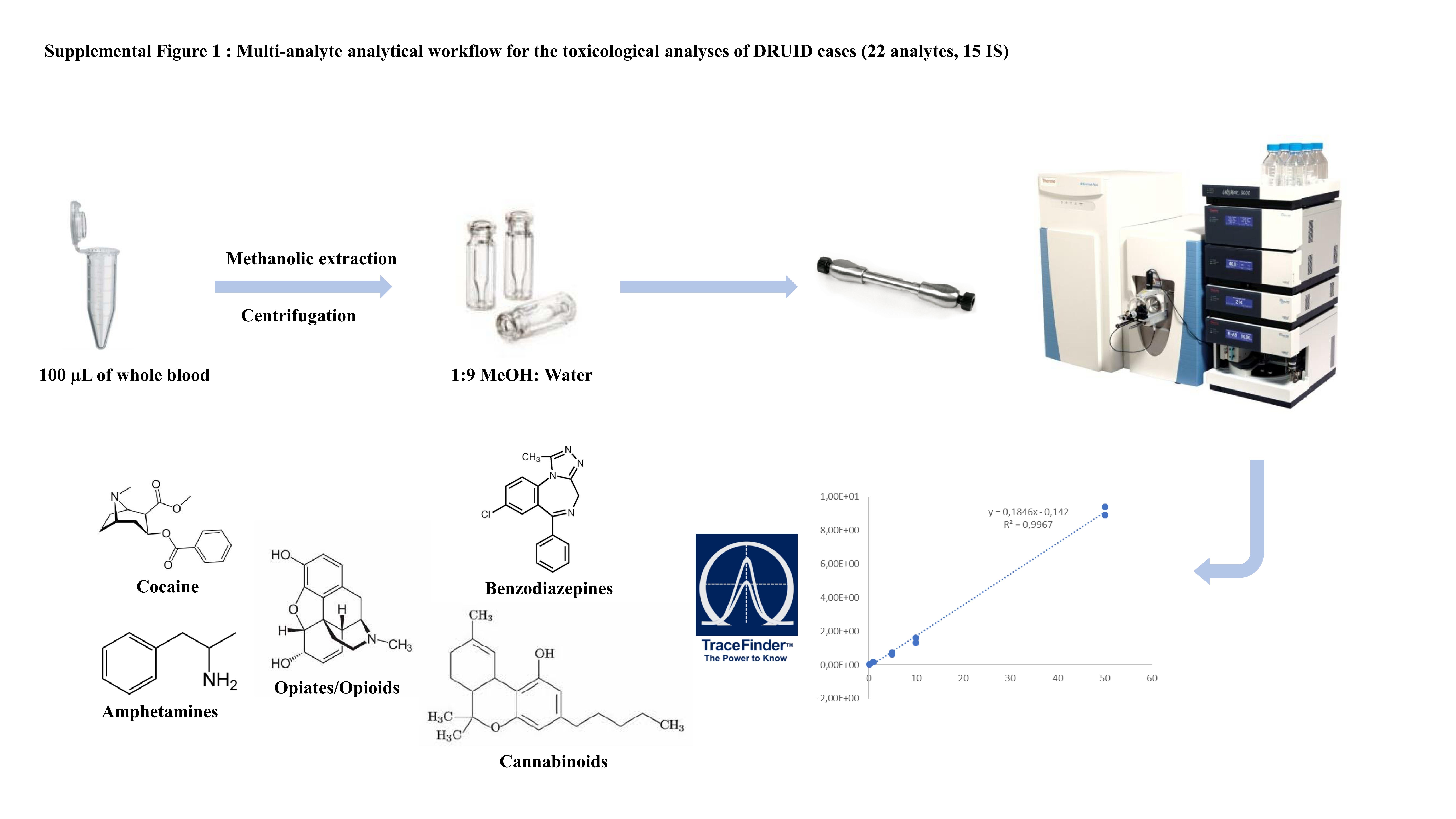

Supplement: Supplementary file 1 [file Image_1.JPEG]
